# Developing an Acoustic Sensing Yarn for Health Surveillance in a Military Setting

**DOI:** 10.3390/s18051590

**Published:** 2018-05-17

**Authors:** Theodore Hughes-Riley, Tilak Dias

**Affiliations:** Advanced Textiles Research Group, School of Art & Design, Nottingham Trent University, Bonington Building, Dryden Street, Nottingham NG1 4GG, UK; tilak.dias@ntu.ac.uk

**Keywords:** electronic textiles, E-textiles, noise induced hearing loss, acoustic trauma, tinnitus, noise exposure, occupational health, occupational noise

## Abstract

Overexposure to high levels of noise can cause permanent hearing disorders, which have a significant adverse effect on the quality of life of those affected. Injury due to noise can affect people in a variety of careers including construction workers, factory workers, and members of the armed forces. By monitoring the noise exposure of workers, overexposure can be avoided and suitable protective equipment can be provided. This work focused on the creation of a noise dosimeter suitable for use by members of the armed forces, where a discrete dosimeter was integrated into a textile helmet cover. In this way the sensing elements could be incorporated very close to the ears, providing a highly representative indication of the sound level entering the body, and also creating a device that would not interfere with military activities. This was achieved by utilising commercial microelectromechanical system microphones integrated within the fibres of yarn to create an acoustic sensing yarn. The acoustic sensing yarns were fully characterised over a range of relevant sound levels and frequencies at each stage in the yarn production process. The yarns were ultimately integrated into a knitted helmet cover to create a functional acoustic sensing helmet cover prototype.

## 1. Introduction

This work created a textile noise dosimeter suitable for military use. Overexposure to noise is known to cause permanent hearing damage, and as a result, employers are required to implement suitable health-monitoring measures when workers will be exposed to loud noises [1,2,3]. Noise exposure can lead to a variety of disorders [4,5,6] with tinnitus and noise-induced hearing loss (NIHL) being the most common among them.

Hearing damage is dependent on the amplitude, frequency and duration of sound exposure. Initially, NIHL results in a loss of hearing at a range of higher frequencies (3–6 kHz) [7] and can hinder the perception of sound. NIHL is a result of either an extremely high-level sound or gradual exposure to high-levels of sound over a period of time. A secondary symptom of acoustic trauma can be tinnitus, which presents as the perception of a sound when no sound source is present. In both cases, damage can be permanent with no accepted common treatment methods. In the last 10 years there have been 1505 claims to the United Kingdom Industrial Injuries Disablement Benefit scheme relating to NIHL in the period 2007–2016 [8], and in the United States of America more than 10 million adults under 70 (6 percent of the under 70 population) exhibit some form of hearing loss from noise exposure [9].

Many military activities generate high levels of noise, which fall into two categories; impulse noise, or background noise. Impulse noise can be related to activities such as firearms use, where a single gunshot is capable of producing a sound pressure level (SPL) of 140–170 dB(A) [7]. While the use of firearms is a major source of noise, research has also investigated sound exposure experienced by helicopter aircrews, who are exposed to continuous noise levels of up to 100 dB(A) during flying operations [10]. The type and magnitude of noise exposure will, therefore, depend on the military role of the personnel involved. A study by Rovig et al. investigated the hearing health of a number of personnel operating on an aircraft carrier flight deck [11]. As part of this study they showed that staff in different roles were exposed to significantly different levels of noise. It was observed that flight-deck personnel had an average exposure of 109 dB(A), compared to 92 dB(A) for engineers.

Overexposure to noise resulted in 278 personnel being discharged from the UK Armed Forces between April 2011 and March 2016 [12]. NIHL is also a serious concern for militaries in other countries, such as the USA [13]. There have been a number of studies into hearing damage specific to the military [14,15], with research showing a far higher number of cases of hearing damage among military personnel when compared to other noise-exposed workers, such as shipbuilders [16]. Reduced sound perception can introduce significant dangers in a military setting, where orders may not be heard correctly. As highlighted by Grantham, NIHL caused communication issues during the Battle of Fallujah [14].

A noise dosimeter is the most reliable way to determine a worker’s noise exposure; however, commercially available solutions are not suitable for military use. The device must not interfere with normal military operations, which may not be true for jacket-mounted devices or helmet attachments currently available on the market. Further to this, asymmetric hearing damage is known to be more common when firearms are frequently used compared to the acoustic injuries encountered in civilian roles [17]. Therefore, in a military setting it is desirable to monitor the noise exposure to each ear individually.

As a result, it was proposed that the dosimeter should be incorporated into a helmet cover, with sensing elements on both sides of the helmet cover. This required the creation of an electronic textile helmet cover.

Electronic textiles and smart textiles have grown in prevalence in recent years finding applications in a number of sectors [18] including energy-storing fabrics to cool buildings [19], energy harvesting [20], and medical devices [21]. Wearable electronics have also seen a recent increase in attention. A number of wearable triboelectric nanogenerators have been developed which are capable of collecting acoustic noise [22,23]; while designed as generators, the same technology could be applied to allow for sensing applications [24], such as acoustic sensing [25,26].

Electronics can be integrated into textiles in a number of ways; by attaching them onto the surface of a fabric, integrating conductive elements into a textile, or incorporating electronics at a yarn level, where the yarns can be used subsequently to create a fabric [27]. Integration at a yarn level hides the electronics better and can protect electronics from external forces better.

In this work, commercially available microelectromechanical system (MEMS) microphones were used as the sensing element of an electronic acoustic sensing yarn, based on Nottingham Trent University’s fibre electronic technology [28,29]. The technology has previously been used to create a temperature-sensing yarn [30]. MEMS devices were used as opposed to a more-complex triboelectric devices to simplify the manufacturing process and reduce cost.

To create the acoustic sensing yarn, microphones were first soldered onto fine multi-strand copper wire, attached to a carrier fibre to increase the tensile strength, and protected with a micro-pod crafted from an ultraviolet (UV)-curable resin to protect the microphone. The ensemble, with additional packing fibres, was then put through a small diameter circular warp knitting machine, covering it with a fibre sleeve to form the acoustic sensing yarn. The final acoustic sensing yarn was drapeable, flexible, and soft to the touch like a normal textile yarn.

While commercial sensing devices (microphones) were employed to construct the acoustic sensing yarn, the inclusion of the micro-pod and fibers may alter the sensor’s response to external stimulus. Textiles structures are commonly used in sound-absorbing applications [31,32,33], as this type of porous structure gives sound waves many opportunities to interact with the fibers and lose energy. Both the structure of the material, and the sound-wave frequency, will affect the sound absorbance properties of a textile [32,33]. It is therefore possible that the knitted textile structure of the acoustic sensing yarn would absorb sound. As it is very difficult to model a textile structure accurately, the sound absorbance of textile had to be determined and understood experimentally.

As a result, the acoustic sensing yarns embedded with microphones were carefully characterized, and design rules for the encapsulation were developed. To achieve this the yarns were tested for a range of relevant frequencies and amplitudes at each stage of the yarn production process (informed by EN 61672-1:2013 [34], and other sources [35]).

Once characterised, the acoustic sensing yarns were paired with self-contained, supporting electronics to record and store the collected acoustic information (herein referred to as the hardware module). The yarns were subsequently re-tested with the hardware module. Finally, the acoustic sensing yarns, and supporting hardware, were incorporated into a knitted helmet cover created using computerised flat-bed 3D knitting technology. Two yarns were integrated into each helmet cover, one over each ear. The completed helmet cover dosimeter was tested over a range of conditions to validate the functionality of the final prototype for its intended purpose.

## 2. Materials and Methods

### 2.1. Acoustic Sensing Yarn Fabrication and Design Considerations

**Figure 1:**
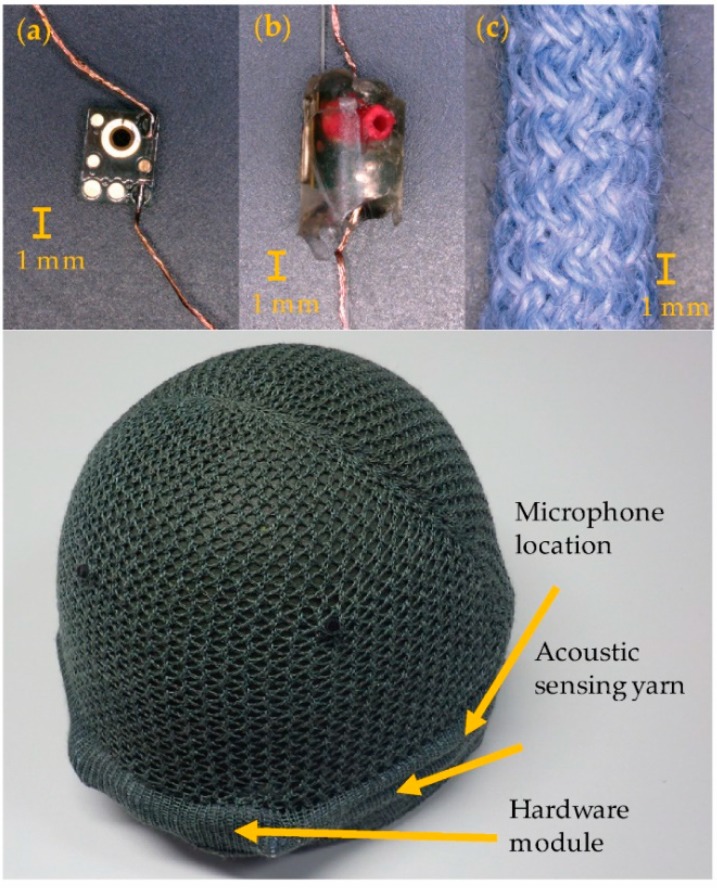
Images at different stages in the production of the prototype acoustic sensing helmet cover. (**a**) Soldered microphone; (**b**) encapsulated microphone; (**c**) final acoustic sensing yarn; (**Bottom**) Photograph of the rear of an acoustic sensing helmet cover prototype. The cover is on a British Army Mk6 helmet. Important components of the cover have been annotated.

The acoustic sensing yarns were fabricated using a handcrafting process in three steps (as shown in Figure 1a–c). Initially, a MEMS microphone was soldered to multi-strand copper wire using a lead-free solder paste (Nordson Solder Plus 7024454 Lead-free; Nordson Corperation, Westlake, OH, USA) and an infrared (IR) spot reflow soldering system (PDR IR-E3 Rework System; PDR Design and Manufacturing Centre, Crawley, UK). The PUI Audio VM1010 (PUI Audio, Dayton, OH, USA) was chosen as the sensing element in this work after extensive trials with other MEMS microphones for this application (not presented in this paper). Ultimately, this microphone was chosen as it was able to operate correctly without external power and because it had a degree of moisture and dust ingress protection [36].

Eliminating the need for an external power source was highly desirable as it significantly simplified the construction process because only two copper interconnects, and not three, were needed. The requirement for external power could be eliminated by wiring the GA1 pin (wake-on-sound acoustic threshold adjust pin 1) to the ground on the recording device. Physically, it was believed that the energy generated by the motion of the piezoelectric element at the core of the microphone (due to incident soundwaves) was providing enough power for the device.

Once soldered, the microphone and solder joints were encapsulated within a UV curable polymer micro-pod, which protected the device from mechanical stresses and provided some chemical resilience. A synthetic yarn with a high-tensile strength (Vectran™, Kuraray America Inc., Houston, TX, USA) was included within the micro-pod to add strength to the ensemble in the direction of the copper interconnects. The micro-pod was formed by injecting resin (Multi-Cure^®^ 9001-E-V-3.7, Dymax Corporation, Torrington, CT, USA) into a cylindrical Teflon mould and curing it using a UV light source (Blue-Wave™ 50, Dymax Corporation, Torrington, CT, USA). To allow the microphones to continue to function, a small cavity in the micro-pod was required over the microphone’s inlet, which was created using a rubber tube (o.d. ~1 mm). To avoid resin entering the cavity and impairing the microphone’s operation, resin was injected and cured in multiple stages.

The micro-pod and copper interconnects were then inserted within a circular warp-knitting machine (RIUS MC braiding machine, RIUS, Barcelona, Spain). Four polyester packing yarns were included around the micro-pod which created a final acoustic sensing yarn of a diameter of 7 mm.

### 2.2. Prototype Acoustic Sensing Helmet Cover

The prototype acoustic sensing helmet covers consisted of three main components: the knitted helmet cover, the acoustic sensing yarns, and the supporting hardware electronics modules. The hardware modules were created using two modified commercially available Dictaphones (Mini USB Voice Recorder, Wjiling), as this offered a low-cost and small size solution. The final hardware modules were covered in a silicon-based coating to add a degree of water-resistance to the modules and improve their mechanical resilience.

The bespoke knitted helmet cover used in this work was designed to fit on top of a standard British Army combat helmet. The cover was produced using a merino wool and Kevlar mix on a Shima Seiki computerised flat-bed knitting machine (SIR, 14 gauge, Shima Seiki, Wakayama, UK). The helmet cover had two knitted channels on either side allowing for the insertion of the acoustic sensing yarns above each ear. These yarns each attached to a hardware module, which was inserted into knitted pockets at the rear of the helmet cover (Figure 1).

### 2.3. Testing Procedure

**Figure 2:**
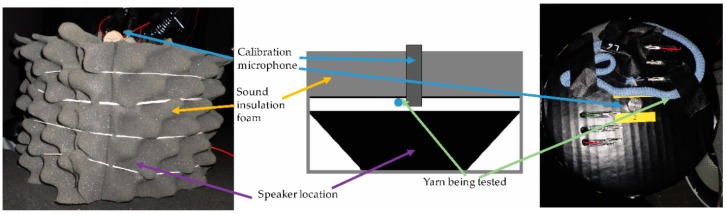
The inner acoustic testing chamber. (**Left**) A photograph of the chamber. (**Middle**) A schematic of the interior of the chamber. (**Right**) A photograph of the lid of the chamber showing the calibration microphone and yarn placements.

Acoustic sensing yarns were tested in an acoustically quiet environment achieved using a bespoke acoustic testing chamber. The testing chamber comprised of two parts: the outer chamber was an acoustically insulated rack designed for storing computer servers (Orion Mini Acoustic Rack, Orion, Leeds, UK), while the inner chamber was built from a 17 cm diameter extruded polyvinyl chloride (PVC) pipe (see Figure 2). The PVC pipe fit over the top of a base speaker which was used to generate the test sounds (Bass Face SPL6M.2 800 W 6.5 inch Mid-Bass Car Speaker Single, Base Face, Macclesfield, UK). The PVC tube was clad in acoustic insulation foam (Adhesive PUR Foam Soundproofing Sheet, RS Components Ltd., Corby, UK) to provide sound insulation. The final inner chamber was 175 mm high. A cap for the inner chamber was built from insulation foam and reinforced with cardboard and a plastic coating. This cap was used to position the microphones under test as well as a high-accuracy calibration microphone used to determine the output sound from the speaker (Brüel & Kjær Type 4190 with a Photon + signal analyser; Brüel & Kjær, Nærum, Denmark). Sounds into the speaker were computer generated and fed through an amplifier (TeLe Hi-Fi A6 audio amplifier, Venezia, Italy).

Generated audio signals had a sinusoidal waveform at a consistent amplitude and frequency; this was confirmed using the calibration microphone. Some additional spectral features were observed near the operating limits of the system (low frequencies and very high amplitudes). Under normal operating conditions, the frequencies between 63 Hz–8000 Hz could be explored without additional (undesirable) spectral features. Additional spectral features at 31.5 Hz prevented accurate testing at that frequency.

The experimental design ensured that a slight misplacement of the lid did not have a significant effect on readings, as the input sound level was determined by the calibration microphone and not a setting on the computer. The chamber minimised the effects of external noise on the microphone pick-up during testing and provided an important layer of protection to the operator when high sound pressure level (SPL) sounds were being tested.

For yarn-level testing, the samples were attached to the top surface of the inner chamber and their copper wires were clipped to wires leading to a signal out and a ground. The signal was run through a 5 m audio extension (Maplin 3.5 mm Stereo Jack Extension Cable 5 m; Maplin Electronics, Rotherham, UK) into a sound card (Dynamode USB Sound Card; Dynamode UK Ltd., Watford, UK).

Signals were recorded and processed using a bespoke Python v2.7 (Python Software Foundation, Wilmington, DE, USA) script, which made use of the SciPy [37], Matplotlib [38] and PyAudio [39] modules. A 10-second signal was recorded (based on information given in British Standard EN 61672-1:2013 [34]), the signal was fast-Fourier transformed, and a peak picking algorithm was used to find peaks between 5 Hz and 10,000 Hz in the signal. The signal peaks were then sorted by amplitude. If the selected frequency was within ±1% of the value identified by the calibration microphone, then the sensor under test was deemed to have correctly selected the frequency.

Peak values were used throughout this work unless otherwise stated. Presented amplitude values were typically the average of five results, with error values given as the standard deviation. The amplitude of the sensors response is given in arbitrary units (arb) related to a voltage output from the microphone.

For prototype-level testing, the yarns, or the yarns within the knitted helmet cover, were attached onto the top surface of the inner acoustic testing chamber. The hardware module was used to record a series of sounds covering a range of frequencies and amplitudes. The recordings were downloaded and 10-second intervals relating to each test condition were extracted (using Audacity v2.1.2). The sound files were individually read into a bespoke Python script that analysed the data in a similar fashion to the process described for the yarn-level testing, output and amplitude value.

Data presented in this work has been prepared using either Microsoft Excel (Microsoft Corporation, Redmond, WA, USA), IGOR Pro (Version 7.0.2.2; Wavematrics, Tigard, OR, USA), or Matplotlib [38].

## 3. Results and Discussion

### 3.1. Acoustic Sensing Yarn Validation

**Figure 3:**
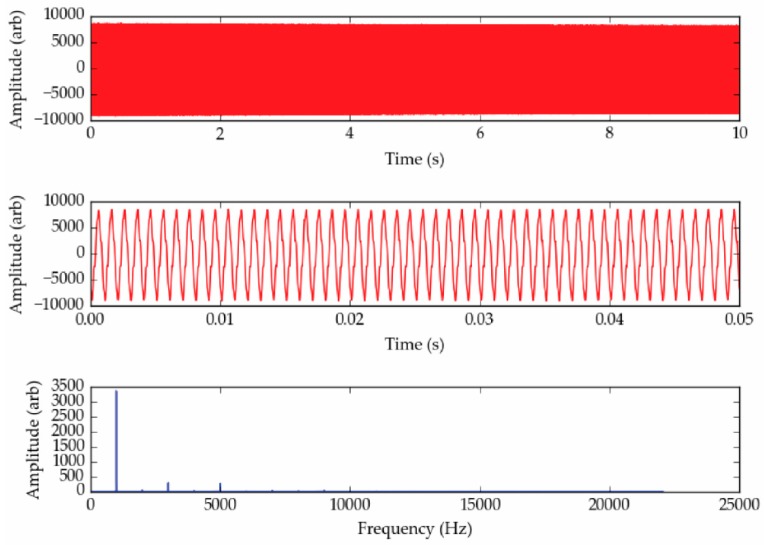
Example data showing the sensor response to a 1000 Hz signal using an acoustic sensing yarn. (**Top**) The raw collected signal. (**Middle**) A graph of the raw signal covering a shorter time interval, showing the waveform. (**Bottom**) The fast-Fourier transform of the signal. In this example, there are some higher frequency spectral features, however the main signal is seen as a 1000 Hz.

Over the course of this investigation eight microphones were examined over a range of relevant frequencies and sound pressure levels (SPL) at different points in the acoustic yarn production process to understand fully how embedding the microphones within a yarn may affect its response. Figure 3 shows and example of the type of data that the microphones collected.

Figure 3 clearly shows a dominant peak in the frequency spectrum. The amplitude of this peak would be recorded and output by the software, along with the frequency of the peak.

**Figure 4:**
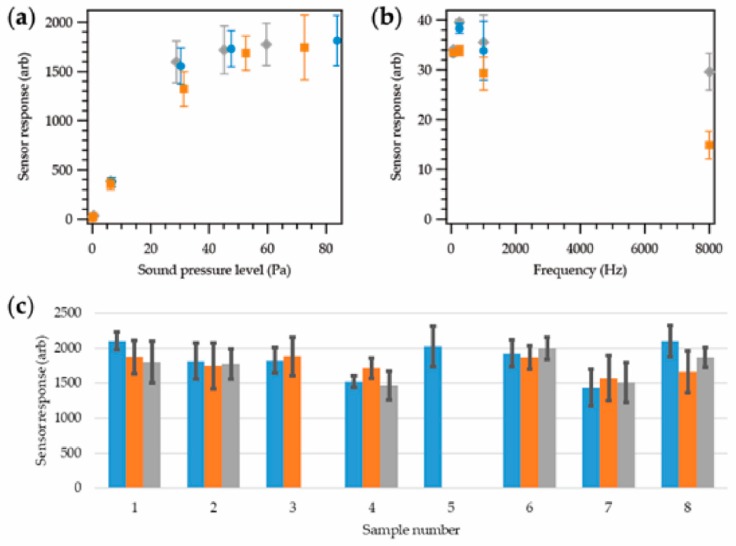
Sensor response for microphones at different stages of encapsulation; soldered to copper interconnects (blue; 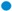), resin encapsulation (orange; 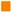), final yarn (grey; 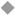). All data points were the average of five repeat measurements. (**a**) Sensor response as a function of sound pressure level at a fixed frequency of 1000 Hz. Tests were carried out between 0.1 and 81.2 Pa (74.8–132.2 dB), with the sensor operating correctly at all amplitudes across this range. (**b**) Sensor response as a function of frequency at a fixed sound pressure level of 0.43 ± 0.02 Pa. Note that the soldered microphone was not able to detect signals at 8000 Hz correctly. (**c**) Sensor response for eight different microphones at different stages of encapsulation. The input sound had an amplitude of 69.2 ± 9.6 Pa with a 1000 Hz frequency. Note that the large variation in the sound pressure level did not matter in this case, as this level was well above the linear range of the sensor (Figure 4a). Within the experimental error, the sensor response appeared to be unaffected by the encapsulation process.

Figure 4a shows the response of a microphone at different stages of encapsulation at different sound pressure levels. Six SPLs between ~75 dB (0.1 Pa) and ~130 dB (63 Pa) were explored; 75 dB (below which human hearing is not damaged at all), ~87 dB (the exposure limit in the UK), ~110 dB, ~124 dB, ~127 dB, and ~130 dB (the limit of the testing apparatus).

Figure 4b shows the response of the same microphone at different frequencies with a fixed SPL of 86.6 ± 0.3 dB. Four frequencies were investigated between 63 Hz and 8000 Hz, which was informed by British Standard (BS) EN 61672-1:2013 [34]. Frequencies at 250 Hz, and 1000 Hz were included to give a good coverage of the relevant octave bands (octave bands 1, 3, 5, and 8 were explored).

Figure 4c shows the responses of the eight microphones at ~130 dB at different stages of encapsulation.

All values are given as a relative arbitrary value (arb) that is related to the voltage output from the sensor. In a practical application, the data in Figure 4 could be used to calibrate the sensor response to a dB (SPL) level. Due to breakages during manufacturing, full data sets at each stage of the production process only exist for six microphones. Each data point was taken as a discrete measurement at a given SPL and frequency.

Figure 4a clearly showed that the different stages of encapsulation had no bearing on the performance of the microphone at different sound pressure levels. The response of the sensor was seen to increase linearly with an increase in the SPL until around 48 Pa. Above ~48 Pa, a flat signal response was observed.

The frequency response of the sensor (Figure 4b) showed a slight increase with frequency, followed by a decrease; however, the relationship was hard to quantify fully given the number of data points collected. The encapsulation process appeared to have a minimal effect on the sensors’ response. The greatest deviation was observed at 8000 Hz. In this case the unencapsulated microphone experiments failed to identify the 8000 Hz signal successfully.

Figure 4c further reaffirmed that the effect of embedding the microphone within a yarn had little effect on the sensor’s response. Within the experimental errors, most of the sensor responses from each microphone were in agreement with one another. This showed that the fiber density and thickness of the covering textile sheath was not sufficient to have a significant dampening effect on the acoustic properties of the sensor, which was an important outcome.

**Figure 5:**
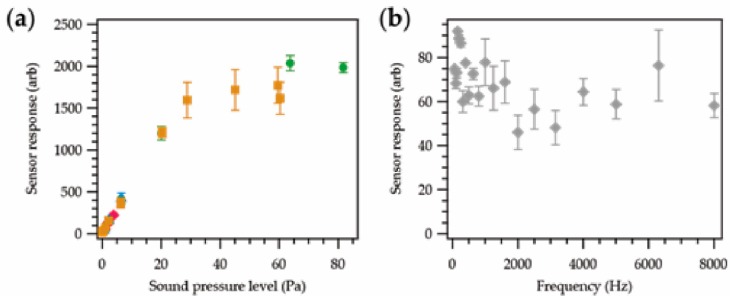
Sensor response for an acoustic sensing yarn. All data points were the average of five repeat measurements. (**a**) Sensor response as a function of sound pressure level at four frequencies, 125 Hz (green; 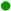), 1000 Hz (yellow; 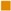), 4000 Hz (blue; 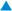), and 8000 Hz (pink; 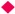). Tests were carried out between 0.02 and 82 Pa, and gave similar responses regardless of frequency. It should be noted that higher SPLs were not possible for all frequencies explored, given the limitations of the testing apparatus. (**b**) Sensor response as a function of frequency at a fixed sound pressure level of 1.03 ± 0.05 Pa.

Having proven that the microphones could be encapsulated within a yarn without having a significant impact on the sensors’ response, more extensive testing was conducted for the final yarn based on British Standard (BS) EN 61672-1:2013 [34]. The amplitude response of a yarn was explored for 125 Hz, 1000 Hz, 4000 Hz, 8000 Hz, at 10 dB increments. If other data-points were available (taken from Figure 4) they were added for completeness. The frequency response of the acoustic sensing yarn was also explored between 63 and 8000 Hz in one-octave increments. Results are shown in Figure 5.

The results in Figure 5a showed the sound pressure level dependence of the sensor response at four different frequencies identified in the British Standard. While 31.5 Hz was also discussed in the standard as a frequency that should be tested, this was not explored given the limitations of the experimental test set-up. The authors do not believe this would be a significant problem given that 31.5 Hz is below the minimum frequency required of a Class 2 sound meter as defined by the standards [34]. In all cases, the sensor gave a similar response regardless of the frequency. This showed that a signal response-to-sound-pressure-level conversion can be applied to the data collected with the acoustic sensing yarn, regardless of the frequency explored.

The frequency dependence of the acoustic sensing yarn over a range of frequencies at a fixed SPL of 94 dB (as described in the British Standard) is shown in Figure 5b. As previously observed, the sensor response increased with frequency, up to 160 Hz in this case, and then decreased slightly. There was a factor of two difference between the highest and lowest sensor responses depending on the frequency used. This indicated that applying a frequency-based sound pressure level correction could be used to improve the accuracy of the data collected.

### 3.2. Acoustic Sensing Helmet Cover Validation

**Figure 6:**
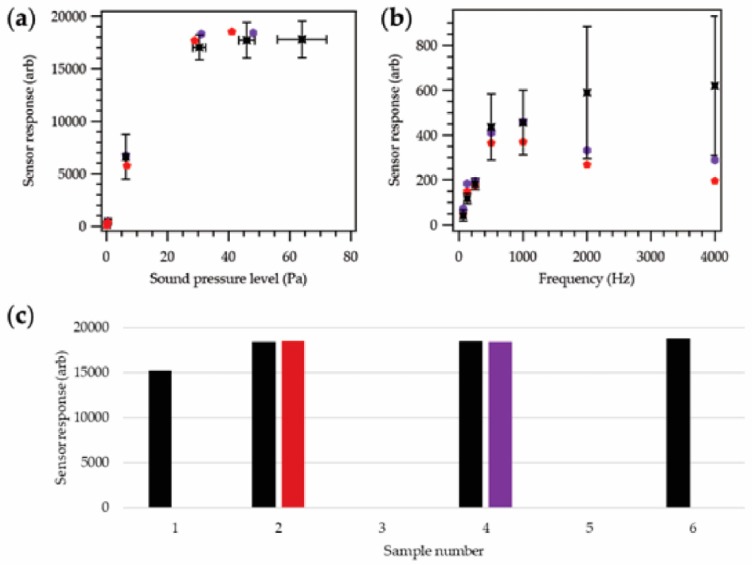
Sensor response for the acoustic sensing yarns where the hardware module was used. Experiments were conducted using the acoustic sensing yarns before (black; 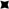) and after (red and purple; 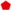, 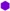) the yarns were inserted within the helmet cover. (**a**) Sensor response as a function of sound pressure level at a fixed frequency of 1000 Hz. Tests were carried out between 0.1 and 8.1 Pa (73.8–112.1 dB), with the sensor operating correctly at all amplitudes across this range. For the acoustic sensing yarn outside of the cover (black), averaged data using four samples is shown. (**b**) Sensor response as a function of frequency at a fixed sound pressure level of 0.43 ± 0.02 Pa. For the acoustic sensing yarn outside of the cover (black), averaged data using four samples is shown. An 8000 Hz signal was also tested; the variation in results at this frequency was very high, and it has been negated from the graph for clarity. Variation in the result when the hardware module was used was also seen to be significant at high frequencies (2000 Hz and 4000 Hz). (**c**) Sensor response for the four acoustic sensing yarns at a fixed input sound of 45.5 ± 3.1 Pa with a 1000 Hz frequency. The sample numbers are consistent with Figure 3.

Following completion of the evaluation of the acoustic sensing yarn, further testing was required to validate the yarn’s use as part of the prototype acoustic sensing helmet cover. Four acoustic sensing yarns were selected for these trials in order to better understand how the responses from the final integrated system might vary between yarns. The experiments were a repeat of those described for Figure 4, except that Figure 6b explored four additional frequencies (125 Hz, 500 Hz, 2000 Hz, and 4000 Hz).

Initially, the yarns were tested with the supporting hardware module outside of the helmet cover. Experiments were then repeated with two of the yarns inserted within the knitted helmet cover (creating the final prototype helmet cover), as shown in Figure 6.

Figure 6a clearly demonstrated the same general sound pressure level to sensor response relationship previously observed; however, the collected (arbitrary) values were higher in all cases; this could be attributed to different amplification electronics within the hardware module compared to the tests when the signals were collected by a soundcard. Signal loss due to the length of cabling between the microphone element and hardware may also account for the difference in values.

Embedding the acoustic sensing yarn within the knitted helmet cover did not appear to affect the yarn’s performance. It should be noted that a 130-dB data point could not be obtained once the yarn was within the helmet cover. This was due to the size of the cover, which prevented it being held in the same position relative to the testing speaker, as with other samples, reducing the maximum amplitude that could be tested.

During the experiments, it became clear that the sensor response as a function of frequency, shown in Figure 6b, was different to that previously observed (Figure 4b and Figure 5b); four additional frequencies were also explored to better understand the behavior. Here, there was a distinct increase in the recorded sensor response with increasing frequency. These values either continued to increase, as seen in the averaged data for the yarns used with the hardware modules, or reduce slightly, as seen with the two yarns in the helmet cover. This was likely due to the properties of the preamplifier within the mobile hardware module. The module was designed as a Dictaphone; while the human voice is typically between 85 and 3400 Hz, in telephony the vocal frequency range is normally limited to 300–3400 Hz. It was possible that this was why a level frequency response below 500 Hz was not observed. Despite the lack of a level frequency response, the observed sensor response-frequency relationship could be used in the processing software to correct measurements.

It is important to note that the 8000 Hz point was not included in this graph given the significant variations observed between different yarns with hardware modules observed at this frequency; where the sensor response at 8000 Hz was seen to be 2809.2 ± 1597.4 arb when the yarns were attached to the hardware module, and 448.6 and 458.7 arb when the yarns were fully incorporated into the helmet cover. There were also some large differences in the results at 2000 Hz and 4000 Hz. These variations were likely due to the limitations imposed by the hardware module as the yarns were previously seen to operate correctly up to 8000 Hz.

Despite this limitation, it should be noted that the 8000 Hz test point was chosen to reflect the British Standard for sound-level meter specifications [34], and the authors do not believe that most high-intensity sounds in a military setting will be at higher frequencies. To confirm this assertion, the authors performed a Fourier analysis on a series of military and gunfire noises available (open source) on the internet. Of the 11 sound files analyzed, the peak frequency was always below 4000 Hz, with most peak frequencies being much lower (below 1000 Hz).

Frequency-dependent effects could also be corrected using the processing software. As previously highlighted, the variations in the results may have been dependent on the supporting hardware module. This was a modified commercial device, and these may have varied from unit to unit or may have been damaged during modification. The two acoustic sensing yarns chosen for integration into the final helmet cover were selected as they exhibited a similar relationship under all testing conditions; from Figure 6a,b, it is clear that their results are in good agreement.

Figure 6c demonstrates that embedding the acoustic sensing yarn within a helmet cover did not have a significant effect on the recorded response at 1000 Hz. This further supported the data presented in Figure 4, showing that a thin knitted sleeve had a negligible dampening effect on an incoming acoustic signal.

A final version of the processing software (not presented) was developed to allow the prototype device to operate for its intended purpose. As well as providing frequency compensation (based on data in Figure 6b), and converting the recorded signals into decibel (SPL) values (using data from Figure 6a), the software also applied an A-weighting filter (defined in the standard [34]), and generated a time averaged noise exposure. The use of an A-weighting filter is common in sound meters and accounts for the loudness of the sound as perceived by the human ear.

While the helmet cover will provide a representative value for the sound intensity near the ears, the actual amplitude of the sound at the eardrum will ultimately also depend on anatomical factors, such as the size and geometry of the ears, due to the head-related transfer functions, which is unique to each person.

Ultimately, this work has demonstrated an acoustic sensing yarn design, which was fully characterised and integrated into a helmet cover with all relevant supporting elements (Technology Readiness Level 4; a prototype validated in a laboratory environment).

## 4. Conclusions

An acoustic monitoring helmet cover has been produced. The device can measure noise exposure using small-scale MEMS microphones at the core of the yarns used to fabricate the cover. The acoustic sensing yarns have been carefully characterized and validated over a range of frequencies and sound pressure amplitudes relevant to a military setting.

This work showed that the inclusion of a small textile structure around a microphone did not have a substantial effect on the recorded signal; hence, the structure did not absorb sound over the range of conditions tested. The inclusion of an additional knitted tube (which the acoustic sensing yarn was fed into), also did not affect the microphone response.

A bespoke (handcraft) manufacturing technique has been developed that facilitates the repeatable fabrication of multiple yarns that provide consistent responses. Future work will transition the production process to an automated or semi-automated method. A helmet cover, supporting electronics, and processing software have been produced and tested with the acoustic sensing yarns to show the utility of the fully integrated acoustic monitoring helmet cover in a laboratory setting (TRL 4).

Ultimately, the use of a modified commercial hardware solution is not scalable, and, within the work reported, the solution employed introduced an additional degree of variation into the results. Future work will investigate the creation of a bespoke hardware module for the storage of data from the acoustic sensing yarns. Combined with a method to more rapidly produce acoustic sensing yarns, this will allow for the production of a larger number of helmet covers which would be necessary to conduct field trials.
